# Real‐life effectiveness of MP‐AzeFlu in Irish patients with persistent allergic rhinitis, assessed by visual analogue scale and endoscopy

**DOI:** 10.1002/iid3.237

**Published:** 2018-10-11

**Authors:** Ranbir Kaulsay, Duc Tung Nguyen, Hans Christian Kuhl

**Affiliations:** ^1^ Bon Secours Consultant Private Clinic, Beacon ENT and Allergy Clinic at Beacon Hospital Dublin Ireland; ^2^ Meda Pharma GmbH & Co. KG (A Mylan Company) Bad Homburg Germany

**Keywords:** azelastine hydrochloride, endoscopy, fluticasone propionate, Ireland, MP‐AzeFlu, persistent allergic rhinitis

## Abstract

**Introduction:**

Most allergic rhinitis (AR) patients have moderate‐to‐severe, persistent disease. Meda Pharma's AzeFlu (MP‐AzeFlu) combines intranasal azelastine hydrochloride (AZE) and fluticasone propionate (FP) in a novel formulation in a single device to treat AR. This prospective, noninterventional study sought to assess the effectiveness of MP‐AzeFlu (one spray/nostril twice daily; 548 µg AZE/200 µg FP daily dose) in relieving AR symptom severity.

**Methods:**

A visual analogue scale (VAS) was used prior to MP‐AzeFlu treatment on days 0, 1, 3, 7, 14, 21, 28, 35, and 42 by 53 persistent AR (PER) patients seen in routine clinical practice in Ireland. An endoscopy was performed on days 0 and 28, and symptoms of edema, discharge, and redness were scored on a three‐point scale (for both nostrils).

**Results:**

Patients using MP‐AzeFlu experienced rapid VAS score reduction from 73.4 mm (standard deviation [SD], 20.3) at Day 0 to 31.5 mm (SD, 25.0) at day 28 (*P *< 0.0001) to 28.1 mm (SD, 24.1) at day 42 (*P *< 0.0001), a 45.3‐mm reduction. On average, patients achieved a clinically relevant VAS score cutoff of 50 mm before Day 7. Total endoscopy score decreased from 7.5 mm (SD, 3.1) at baseline to 3.5 mm (SD, 2.5) at Day 28. The incidence of severe edema on endoscopy decreased from 53.1% at baseline to 3.8% at Day 28. A similar reduction in the incidence of thick/mucousy discharge (from 28.3% to 4.8%) and severe redness (from 34.9% to 0%) was also observed.

**Conclusions:**

MP‐AzeFlu provided effective, rapid control of PER as assessed by VAS in a real‐world clinical setting in Ireland. Symptom improvement was observed at Day 1, sustained for 42 days, and associated with improved mucosal appearance after 28 days. These results confirm the safety of MP‐AzeFlu and exceed the efficacy demonstrated in phase 3 clinical studies for controlling AR in PER patients.

## 1 INTRODUCTION

Affecting approximately 24% of the European population,^1^ allergic rhinitis (AR) is a highly prevalent allergic respiratory disease with symptoms that include sneezing, nasal obstruction, and mucous discharge.^2^ AR is a long‐term, sometimes lifelong condition that can bother patients for two or more seasons of the year.^1^ AR that is present for ≥4 days/week and ≥4 weeks/year is referred to as persistent AR (PER). AR symptoms can negatively impact patients’ daily activities as well as continually interfere with their work and social lives.^1^, ^2^ Symptoms often lead to sleep deprivation, which takes a toll on those with AR; patients report having trouble falling asleep, waking during the night, and not feeling rested after a night's sleep.^1^

Despite instituting environmental measures to control allergy symptoms, such as use of hypoallergenic household goods as well as replacing old furniture and carpets, most PER patients take medications to prevent and/or control their AR symptoms.^1^ However, allergy symptoms and treatment relief can be difficult to assess accurately due to daily fluctuation in allergen exposure. As a result, a recent guideline from MACVIA‐ARIA (Contre les Maladies Chroniques pour un Vieillissement Actif–AR and its Impact on Asthma) endorses a simple visual analogue scale (VAS) to assess AR control and guide treatment decisions.^2^, ^3^ The VAS is a rapid and reproducible method to measure an individual's disease severity.^4^

MACVIA‐ARIA recommends that treated PER patients with VAS scores of 50–100 mm escalate their treatment to intranasal corticosteroids or intranasal corticosteroids plus azelastine hydrochloride.^3^ MP‐AzeFlu (Dymista®) delivers an intranasal corticosteroid (fluticasone propionate) and an intranasal antihistamine (azelastine hydrochloride) in a novel formulation provided in a single device.^5^ MP‐AzeFlu is indicated for the relief of symptoms of moderate‐to‐severe seasonal and perennial AR (SAR and PAR) if monotherapy with either intranasal antihistamine or glucocorticoid is not considered sufficient.^3^, ^5^

The purpose of this noninterventional study was to evaluate the effectiveness of MP‐AzeFlu nasal spray in routine clinical practice in patients with PER living in Ireland. Effectiveness was assessed by VAS, endoscopy, and sleep quality.

## 2 METHODS

### 2.1 Study design

This prospective noninterventional study (NIS) was conducted at a single center in Ireland to quantify the personal symptomatic burden of PER prior to treatment with MP‐AzeFlu. Eligible patients were adults and adolescents (≥12 years) with moderate‐to‐severe PER who were prescribed MP‐AzeFlu (one spray per nostril twice daily) according to the summary of product characteristics.^5^ PAR diagnosis was verified by local standard practice such as skin prick test or serum‐specific immunoglobulin E measures. The decision to include a patient in the study was made independently from and after the decision to prescribe MP‐AzeFlu nasal spray. The study involved two visits, baseline and follow‐up, scheduled approximately 28 days apart. The study was performed in accordance with European (EU 2001; ICH E2E 2004; EMA 2012) regulations; the study documents were approved by a central ethics committee. At the inclusion/baseline visit, the patient or responsible caregiver completed the informed consent paperwork and the physician documented patient data (demographic information, first diagnosis of PER, and allergen types), symptoms (from patient's card), and previous AR treatments (including current immunotherapy) in an electronic case report form (eCRF).

The intended observation of treatment was 42 days per patient. Patients recorded data on symptom severity, level of disease control, and assessment of sleep on a data collection card, which was sent back by mail to the physician after 6 weeks or returned to the physician at an additional, optional follow‐up visit (the third visit). After receipt of the patient's card, the physician transcribed the information recorded on the patient's card into the eCRF.

### 2.2 Assessments

#### 2.2.1 Visual analogue scale

Patients assessed symptom severity using a 100‐mm VAS ranging from 0 mm (“not at all bothersome”) to 100 mm (“very bothersome”) on Days 0, 1, 3, 7, 14, 21, 28, 35, and 42. Assessments were made in the morning, prior to MP‐AzeFlu use, and reflected the severity of the symptoms experienced in the previous 24 h. The VAS recorded at the inclusion visit reflected the severity of the symptoms that had been experienced in the preceding 4 weeks.

#### 2.2.2 Disease control

On the day after the start of treatment, patients assessed the level of disease control achieved within the previous 24 h using a four‐category scale provided on the patients’ card (symptoms well controlled, symptoms partly controlled, symptoms uncontrolled, or unknown).

#### 2.2.3 Sleep quality

On Days 7, 14, 21, 28, 35, and 42, patients rated their sleep for the previous seven nights on a five‐point rating scale (very good, good, fair, bad, or very bad).

#### 2.2.4 Nasal mucosa

An endoscopy was performed on Days 0 and 28 to evaluate the patients’ nasal mucosa. Edema, discharge, and redness were scored on a three‐point scale for both nostrils.

#### 2.2.5 Safety

The incidence of adverse events (AEs) associated with the use of MP‐AzeFlu was monitored during the course of the study. An AE was defined as any untoward medical occurrence in a patient administered MP‐AzeFlu; it did not necessarily have a causal relationship with the treatment. A serious AE was classified as an event that resulted in death; was life‐threatening; required inpatient hospitalization or prolongation of existing hospitalization; or resulted in persistent or significant disability or incapacity, a congenital anomaly/birth defect, or other medically important conditions. In addition, all suspected adverse drug reactions (ADRs) and special situations were documented by the physician in the eCRF. ADRs were defined as responses to MP‐AzeFlu that were considered noxious and unintended. ADRs were coded using the Medical Dictionary for Regulatory Activities (MedDRA) coding system (version 19.0). Possible special situations included pregnancy, breastfeeding, adverse reaction related to occupational exposure, lack of efficacy or any overdose, abuse, off‐label use, misuse, or medication error. The physician assessed each record for the causality of the event during the process of ADR recording.

### 2.3 Statistical analysis

Patient baseline characteristics were summarized using descriptive statistics. All analyses were based on the safety population, which included all patients who were treated with MP‐AzeFlu and whose data were confirmed by the investigator. Baseline‐adjusted analysis of covariance (ANCOVA) for repeated measurements was used to assess the change in severity of AR based on the difference in VAS measurements from baseline over time. Only patients from the safety population with at least one valid post‐baseline assessment recorded in their diary card were analyzed via ANCOVA. Statistical analyses were performed using SAS® (SAS Institute Inc., Cary, NC, USA) version 9.4 or higher.

## 3 RESULTS

### 3.1 Patient characteristics

Fifty‐three adults and adolescents in Ireland with moderate‐to‐severe PER who were prescribed MP‐AzeFlu by a physician were enrolled in the study and considered the safety population for statistical analysis (Table 1). The mean age of the study population was 31.2 ± 15.2 years (median, 31 years; range, 12‐69 years). The mean treatment duration with MP‐AzeFlu was 33.5 ± 7.3 days (median, 28 days; range, 14‐42 days).

**Table 1:** Baseline characteristics of PER patients in Ireland (*N* = 53)

Characteristic	*N* = 53
Sex, *n* (%)	
Male	26 (49.1)
Female	27 (50.9)
Age, *n* (%), *y*	
12‐17	12 (22.6)
18‐65	40 (75.5)
>65	1 (1.9)
AR history, mean (SD), *y*	13.3 (12.4)^a^
Phenotype, *n* (%)	
PAR only	24 (45.3)
SAR and PAR	29 (54.7)
Number of ARIA criteria, *n* (%)	
1	2 (3.8)
2	3 (5.7)
3	12 (22.6)
4	36 (67.9)
VAS score, mm, mean (SD)^b^	73.4 (20.3)
Patients using ≥2 therapies, *n* (%)	17 (32.1)
Patients using immunotherapy currently or in the past, *n* (%)	6 (11.3)

^a^ *n* = 46.

^b^ The severity of AR ranged from 0 (not troublesome at all) to 100 (intolerable).

AR, allergic rhinitis; ARIA, Allergic Rhinitis and its Impact on Asthma; PAR, perennial AR (allergy to at least one nonpollen allergen [dust mites, animal dander, and/or mold] but no pollen allergens); PER, persistent AR; SAR and PAR, allergy to at least one pollen allergen and at least one nonpollen allergen; SD, standard deviation; VAS, visual analogue scale.

#### 3.1.1 Severity of AR

According to the ARIA classification, moderate‐to‐severe AR is present if at least one of the four criteria (troublesome symptoms; impairment of daily activities, leisure, and/or sport; impairment of school or work; or sleep disturbance) is met. Among the 53 patients, 36 (67.9%) met all four criteria, 12 (22.6%) fulfilled three criteria, 3 (5.7%) satisfied two criteria, and 2 (3.8%) met one criterion. Specifically, daily activities, leisure, and/or sport were impaired in almost all patients (*n* = 51, 96.2%), school or work was impaired in 49 patients (92.5%), and 47 patients (88.7%) had bothersome symptoms. Sleep disturbance was reported by 41 patients (77.4%).

#### 3.1.2 Treatment history

In the year prior to the study period, almost all enrolled patients (*n* = 51, 96.2%) were treated with at least one oral and/or intranasal antihistamine. Oral antihistamine was the most frequently used previous treatment for symptomatic AR (*n* = 48, 90.6%). Other frequently used treatments included intranasal antihistamine (*n* = 11; 20.8%), intranasal decongestant (*n* = 9, 17.0%), and intranasal and systemic corticosteroids (*n* = 5 for each, 9.4%). Additional treatments were used by fewer than 5% of patients. Due to the severity of symptoms, some patients (*n* = 17, 32.1%) employed two or more therapies to control their AR, while others (*n* = 6, 11.3%) had undergone immunotherapy in the past or were undergoing concomitant immunotherapy.

### 3.2 Assessments

#### 3.2.1 Visual analogue scale

**Figure 1:**
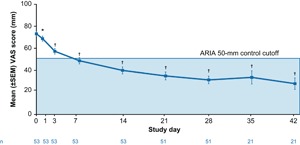
Mean VAS score following MP‐AzeFlu use by PER patients in Ireland (*N* = 53). *P = 0.0106 versus Day 0. ^†^ *P *< 0.0001 versus Day 0. ARIA, allergic rhinitis and its impart on asthma; MP‐AzeFlu, Meda Pharma's AzeFlu; PER, persistent allergic rhinitis; SEM, standard error of the mean; VAS, visual analogue scale

At baseline (day 0, assessment of symptoms experienced during last 24 h prior to start of treatment), the mean VAS score in the total population was 73.4 (standard deviation [SD], 20.3 mm; median, 76.0 mm; range, 12‐100 mm). Mean VAS scores decreased during the treatment period, with the most rapid decrease occurring during the first week of treatment. On average, patients achieved the ARIA‐defined VAS score cutoff of 50 mm for controlled symptoms before Day 7 (Figure 1). MP‐AzeFlu patients continued to experience a rapid VAS score reduction from baseline to 31.5 mm (SD 25.0) at day 28 (*P *< 0.0001) to 28.1 mm (SD 24.1) at day 42 (*P *< 0.0001), an overall reduction from baseline of 45.3 mm. Based on the change from baseline VAS scores, symptoms were significantly improved compared with baseline, and the extent of symptom improvement increased with duration of treatment.

**Figure 2:**
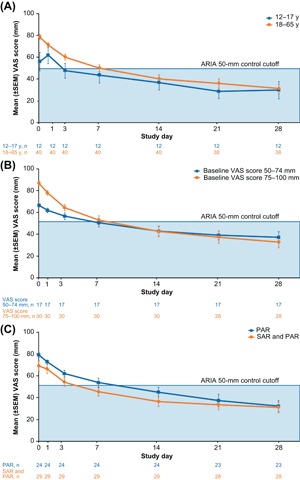
Mean VAS score reduction following MP‐AzeFlu use by PER patients in Ireland (*N* = 53) according to (A) patient age, (B) baseline severity, and (C) phenotype classification. ARIA, allergic rhinitis and its impart on asthma; MP‐AzeFlu, Meda Pharma's AzeFlu; PER, persistent allergic rhinitis; SAR and PAR, allergy to at least one pollen allergen and at least one nonpollen allergen; SEM, standard error of the mean; VAS, visual analogue scale

VAS reductions were consistently seen irrespective of age group (12‐17 y or 18‐65 y; Figure 2A), baseline disease severity (baseline VAS score 50‐74 mm [less severe] or 75‐100 mm [more severe]) (Figure 2B), or traditional AR phenotype classification (PAR only or SAR and PAR) (Figure 2C). Despite some differences in baseline severity score, mean severity scores on the last day were comparable between subgroups, indicating a similar level of symptom control. Due to high proportion of missing diary data on Days 35 and 42 (60.4% on both days), subgroup data are presented only through Day 28.

#### 3.2.2 Disease control after 1 day of treatment

On the next morning after starting treatment, three patients (5.7%) taking MP‐AzeFlu reported that their AR symptoms during the last 24 h were well controlled, 27 patients (50.9%) indicated that their symptoms were partly controlled, and 23 patients (43.4%) rated their symptoms as uncontrolled. Subpopulation (age and disease severity) comparisons revealed similarities between groups.

The results of the symptom control assessment were similar in the age subpopulations (12‐17 years [adolescents; *n* = 12] and 18‐65 years [adults; *n* = 40]). Well‐controlled or partly controlled symptoms were reported in two patients (16.7%) and six patients (50.0%), respectively, in the adolescent subpopulation and 1 patient (2.5%) and 21 patients (52.5%), respectively, in the adult subpopulation. The proportion of patients with uncontrolled symptoms after 1 day of treatment was slightly lower in the adolescent subpopulation (*n* = 4, 33.3%) than the adult subpopulation (*n* = 18, 45.0%).

For the subpopulations by type of AR, no PAR‐only patients had well‐controlled symptoms and 12 of 24 PAR‐only patients (50%) had partly controlled symptoms. In patients with both SAR and PAR (*n* = 29), 3 patients (10.3%) had well‐controlled symptoms and 15 patients (51.7%) had partly controlled symptoms. The proportion of patients with well‐ or partly controlled symptoms after 1 day of treatment was slightly higher in the SAR and PAR population (*n* = 18, 62.1%) than the PAR‐only subpopulation (*n* = 12, 50.0%).

Youden index analyses were conducted to evaluate how well AR symptom severity VAS scores on Day 1 were able to differentiate between symptom control categories.^6^ The estimated optimal VAS cutoff scores were 44 mm for differentiating between well‐controlled and partly controlled/uncontrolled symptoms and 59 mm for differentiating between well‐/partly controlled and uncontrolled symptoms. The corresponding Youden indices of 0.940 and 0.323 indicated high (0.940) and moderate (0.323) accuracy of the determination of the cutoffs. Using these cutoffs, at least 75% of the patients had their symptoms well controlled (median, 43 mm) on the last day.

#### 3.2.3 Sleep quality

**Figure 3:**
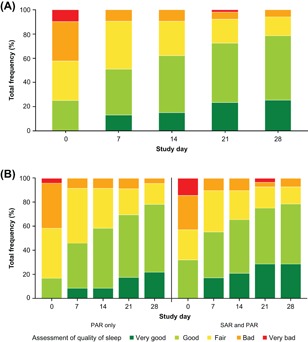
Ratings for quality of sleep from Days 0 to 28 for (A) the total population with available data and (B) allergy type subpopulations with available data. PAR, perennial allergic rhinitis (allergy to at least one nonpollen allergen [dust mites, animal dander, and/or mold] but no pollen allergens); SAR and PAR, allergy to at least one pollen allergen and at least one nonpollen allergen

Patients reported improved sleep quality from baseline through Day 42. Based upon the available data, the percentage of patients with very good or good sleep quality increased from 25.0% (13 of 52 patients) on Day 0 to 78.4% (40 of 51 patients) on Day 28 (Figure 3A). Among the 21 patients who provided data for Days 35 and 42, the proportion with very good or good sleep quality increased further to 81.0% (*n* = 17) at Day 35 and 85.7% (*n* = 18) on Day 42. From Day 7 to 42, no patients reported their sleep quality as very bad.

Improvements in quality of sleep ratings were similar in the PAR‐only and SAR and PAR subpopulations compared with the time course in the total population (Figure 3B). The proportion of patients reporting good or very good sleep quality increased from 16.7% (4 of 24 patients) on Day 0 to 78.3% (18 of 23 patients) on Day 28 in the PAR‐only subpopulation and from 32.1% (9 of 28 patients) on Day 0 to 78.6% (22 of 28 patients) on Day 28 in the SAR and PAR subpopulation. Conversely, the overall rate of patients with bad or very bad sleep quality decreased from 41.7% (10 of 24 patients) on Day 0 to 4.3% (1 of 23 patients) on Day 28 in the PAR‐only subpopulation and from 42.9% (12 of 28 patients) to 7.1% (2 of 28 patients) in the SAR and PAR subpopulation.

#### 3.2.4 Nasal mucosa

Using endoscopy, the physician assessed the presence and intensity of edema, discharge, and redness of the nasal mucosa in both nostrils on Day 0 (*N* = 53) and Day 28 (*n* = 52). After taking MP‐AzeFlu, improvements were noted for all three assessments (Table 2).

**Table 2:** Endoscopy assessment for edema, discharge, and redness

	Left nostril		Right nostril	
Assessment, %	Day 0	Day 28	Day 0	Day 28
Edema				
Absent	5.7	26.4	5.7	28.3
Mild	37.7	67.9	45.3	66.0
Severe	56.6	3.8	49.1	3.8
Discharge				
None	22.6	60.4	20.8	60.4
Clear and thin	47.2	32.1	52.8	34.0
Thick and mucousy	30.2	5.7	26.4	3.8
Redness				
Absent	13.2	41.5	13.2	45.3
Mild	49.1	56.6	54.7	52.8
Severe	37.7	0.0	32.1	0.0

**Figure 4:**
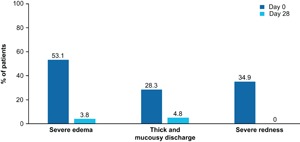
Proportion of Irish PER patients with severe mucosal edema, thick/mucousy discharge, or severe redness before and after 28 days of treatment with MP‐AzeFlu (*N* = 53). MP‐AzeFlu, Meda Pharma's AzeFlu; PER, persistent allergic rhinitis

Overall, after MP‐AzeFlu treatment, the total endoscopy score declined from 7.5 (SD, 3.1) at baseline to 3.5 (SD, 2.5) at Day 28 (*P *< 0.0001). The proportion of patients with severe edema on endoscopy (weighted mean of both nostrils) was reduced from 53.1% at baseline (*N* = 53) to 3.8% at Day 28 (*n* = 52). A similar reduction in the incidence of thick/mucousy discharge (from 28.3% to 4.8%) and severe redness (from 34.9% to 0%) was also observed (Figure 4).

### 3.3 Safety

MP‐AzeFlu was well tolerated by most patients. Among the 53 enrolled patients, a total of two ADRs were reported by two patients (3.8%), and both ADRs were considered possibly related to the treatment by the reporting physician. One patient reported fatigue while the other reported sedation. No serious ADRs were recorded.

## 4 DISCUSSION

The data collected in this NIS represent the background, symptoms, previous treatments, and treatment effectiveness of MP‐AzeFlu for patients in Ireland suffering from PER. Although the intended population for the Irish cohort was calculated to be a maximum of 100 patients, the 53 enrolled patients were considered sufficient to provide an accurate representation of the Irish PER patient population as well as of the safety and effectiveness of treatment with MP‐AzeFlu nasal spray in routine clinical practice. In accordance with the label,^5^ all participating patients were prescribed MP‐AzeFlu because alternative therapies had not been sufficient in the past and/or were considered insufficient to treat acute symptoms.

As in noninterventional MP‐AzeFlu studies conducted in other European countries,^6^, ^7^, ^8^, ^9^ all patients reported moderate‐to‐severe AR according to the ARIA classification. More than half of the patients (54.7%) suffered from SAR and PAR, while the remaining patients were diagnosed with PAR only (45.3%). The proportion of phenotypes differed from a multinational European study of 2988 patients with moderate‐to‐severe AR who used MP‐AzeFlu,^9^ in which 40.1% of patients had SAR, 36.3% of patients had SAR and PAR, 11.8% had PAR only, and 11.8% had an unknown AR type. Despite having more persistent AR symptoms, the enrolled patients in Ireland had a history of AR similar to that of the multinational group (13.3 years vs 12.1 years).^9^

The severity of AR symptoms decreased by a mean of 44 mm on a VAS between the start of treatment with MP‐AzeFlu and the last day of the observational period (on average, 33.5 days). This reduction was similar to that reported by patients with diary documentation for the full 42‐day observational period (mean reduction, 42 mm). Earlier real‐world studies in patients with moderate‐to‐severe AR found mean reductions in VAS score over approximately 14 days of 31,^8^ 50,^9^ 54,^6^ and >60 points,^7^ demonstrating a consistent pattern of substantial improvement in symptoms with MP‐AzeFlu therapy. These findings are consistent with earlier clinical trials of MP‐AzeFlu that found significantly greater reductions in nasal symptoms, including nasal congestion, with MP‐AzeFlu than with FP alone.^10^, ^11^

Prior studies^12^, ^13^ have suggested a >23 mm difference for clinically meaningful changes in VAS scores. In the present study, this threshold was observed after 3 days of treatment in more than half of the patients, as the VAS score decreased by a median of 13 mm. On the last day of treatment (or last diary documentation), 40 of the 53 patients (75%) had a clinically significant decrease in VAS scores from baseline of more than 22 mm (upper quartile −22 mm).

In previous real‐world studies, 50% to >80% of patients with AR had well‐controlled symptoms by approximately Day 14,^6^, ^7^, ^8^, ^9^ consistent with the findings of this study. In a double‐blind clinical trial of MP‐AzeFlu among patients with moderate‐to‐severe SAR, 18% of patients treated with MP‐AzeFlu experienced complete or near‐complete nasal symptom resolution after 14 days.^10^ Therefore, it appears that symptom control with MP‐AzeFlu in the real world may be even greater than that found in clinical studies. As AR is associated with a substantial burden on patients’ quality of life,^14^, ^15^, ^16^, ^17^, ^18^, ^19^ the large proportion of patients experiencing full control of their AR symptoms in real‐world studies suggests that treatment with MP‐AzeFlu may also improve patient outcomes.

Symptom improvement was seen in all patient subpopulations irrespective of type of AR (PAR only, SAR, and PAR), age (adolescents, adults), baseline symptom severity, or sex. Some differences in baseline severity score between subgroups were noted (e.g., patients with PAR only had higher baseline scores than patients with SAR and PAR, and adults had higher baseline scores than adolescents). However, the mean severity scores on the last day were comparable between subgroups, indicating similar levels of symptom control achieved.

Sleep quality improved continuously throughout the study, as reflected by increasing proportions of patients reporting good or very good sleep quality and decreasing proportions reporting fair, bad, or very bad sleep quality. Similar results were obtained for the PAR‐only and SAR and PAR subpopulations. Although some differences in data reporting and baseline characteristics were noted, the results of this study are consistent with the findings of the same NIS conducted in Sweden.^20^ Sleep impairment has been well established as a debilitating symptom associated with AR2^1^, ^2^, ^3^, ^4^, ^5^, ^6^, ^7^, ^8^, ^9^, ^10^, ^11^, ^12^, ^13^, ^14^, ^15^, ^16^, ^17^, ^18^, ^19^, ^20^, ^21^, ^22^, ^23^, ^24^, ^25^, ^26^; therefore, the improvement in sleep quality with MP‐AzeFlu observed in these real‐world studies has the potential to greatly improve AR patients’ quality of life.

Nasal endoscopy revealed that the presence and severity of edema, redness, and discharge in both nostrils distinctly decreased during 4 weeks of treatment with MP‐AzeFlu. Accordingly, the rate of patients with severe redness or edema decreased from about one‐third to no or only a few individuals. Similar results were obtained for nasal discharge. These results are consistent with previously reported findings that MP‐AzeFlu reduces inflammatory markers in an in vitro model of human nasal inflammation.^27^ The present study demonstrated that during treatment with MP‐AzeFlu, improvement of subjective symptoms (AR severity by VAS and sleep quality) was consistent with reduction of objective symptoms (edema, redness, and discharge in both nostrils). In this sense, the endoscopy findings can be taken as visual evidence of the pathophysiological changes underlying patients’ reported symptom improvement.

PER is a distinct category of AR that merits special attention in the clinic, as patients with PER are at higher risk for sequelae including asthma.^2^, ^28^ Topical treatments are recommended for PER,^2^ in part to avoid the long‐term safety concerns associated with systemic medications. In this context, our finding that MP‐AzeFlu is effective in patients with PER may help establish this newer option as a preferred treatment for patients with PER. A long‐term clinical trial of MP‐AzeFlu established its efficacy^11^ and safety^29^ over 52 weeks of treatment, and the current study reinforces the appropriateness of MP‐AzeFlu for treatment of patients with persistent AR symptoms. Optimal pharmacotherapy for PER will not only control symptoms but also help patients function better (via improved sleep) and improve their quality of life. The findings of this study, which add to the body of evidence of previous real‐world and clinical studies of MP‐AzeFlu,^6^, ^7^, ^8^, ^9^, ^10^, ^11^, ^29^, ^30^, ^31^ further support MP‐AzeFlu as a treatment with broad benefits for this patient population.

As with any study, there were limitations associated with this analysis. The noninterventional and observational study design lacked a control group and/or randomization scheme. Also, missing data could be a source of bias when analyzing clinical study data. The last day of treatment or documentation in the diary card was on average 33.5 days (median, 28 days) after the start of the treatment (Day 0), which was less than the planned 42‐day observational period. The high proportion of missing diary data on Days 35 and 42 suggests that patients may have misunderstood that the diary entries for weeks 5 and 6 were to be returned via mail, not at a regular visit. Alternatively, many of the patients who responded well to therapy may have deemed follow‐up after Day 28 unnecessary.

Among the effectiveness variables, severity of AR by VAS score and sleep quality had the most missing data on Days 35 and 42 (*n* = 32; 60.4%). Until Day 28, however, the amount of missing diary data was low (<4%). For severity of AR by VAS and sleep quality, the time course of completers and dropout patients was very similar to the time course of all patients until Day 28. In addition, the reported results remained stable up to Days 35 and 42. Finally, values obtained for Day 42 and the last day of documentation were very similar, indicating that the time course was not biased by selective loss of patients (i.e., based on lack of effectiveness).

The two reported ADRs, sedation (*n* = 1) and fatigue (*n* = 1), are not unique to this study, as they are in line with what is mentioned in MP‐AzeFlu labeling.^5^ The safety results of this study are generally consistent with the product labeling, and no other ADRs occurred in this small study population.

In conclusion, MP‐AzeFlu provides effective and rapid control of PER as assessed by VAS in a real‐world clinical setting in Ireland. Symptom improvement was observed at Day 1, was sustained for 42 days, and was associated with improved mucosal appearance after just 28 days. These results confirm the safety of MP‐AzeFlu and exceed the efficacy demonstrated in phase 3 clinical studies for controlling AR in PER patients.

## CONFLICTS OF INTEREST

RK has received honoraria from ALK‐Abello, GlaxoSmithKline, Menarini Group, Novartis, and Schering‐Plough as well as educational and travel grants from Meda (a Mylan company). DTN and HCK are employees of Meda and have no other conflicts of interest.
